# A putative high affinity phosphate transporter, CmPT1, enhances tolerance to Pi deficiency of chrysanthemum

**DOI:** 10.1186/1471-2229-14-18

**Published:** 2014-01-10

**Authors:** Peng Liu, Sumei Chen, Aiping Song, Shuang Zhao, Weimin Fang, Zhiyong Guan, Yuan Liao, Jiafu Jiang, Fadi Chen

**Affiliations:** 1 College of Horticulture, Nanjing Agricultural University, Nanjing 210095, China; 2 Jiangsu Province Engineering Lab for Modern Facility Agriculture Technology & Equipment, Nanjing 210095, China

**Keywords:** *Chrysanthemum morifolium*, *CmPT1*, Functional characterization, Complementation assay, Transgenic plants

## Abstract

**Background:**

Inorganic phosphate (Pi) is essential for plant growth, and phosphorus deficiency is a main limiting factor in plant development. Its acquisition is largely mediated by Pht1 transporters, a family of plasma membrane-located proteins. Chrysanthemum is one of the most important ornamental plants, its productivity is usually compromised when grown in phosphate deficient soils, but the study of phosphate transporters in chrysanthemum is limited.

**Results:**

We described the isolation from chrysanthemum of a homolog of the *Phosphate Transporter 1* (*PT1*) family*.* Its predicted product is a protein with 12 transmembrane domains, highly homologous with other high affinity plant Pi transporters. Real-time quantitative PCR analysis revealed that the gene was transcribed strongly in the root, weakly in the stem and below the level of detection in the leaf of chrysanthemum plants growing in either sufficient or deficient Pi conditions. Transcript abundance was greatly enhanced in Pi-starved roots. A complementation assay in yeast showed that CmPT1 partially compensated for the absence of phosphate transporter activity in yeast strain MB192. The estimated *K*_*m*_ of CmPT1 was 35.2 μM. Under both Pi sufficient and deficient conditions, transgenic plants constitutively expressing *CmPT1* grew taller than the non-transformed wild type, produced a greater volume of roots, accumulated more biomass and took up more phosphate.

**Conclusions:**

*CmPT1* encodes a typical, root-expressed, high affinity phosphate transporter, plays an important role in coping Pi deficiency of chrysanthemum plants.

## Background

After nitrogen, phosphorus is the most frequently limiting plant macronutrient [1]. Despite its abundance in the soil, most of it is present in a form which is not readily accessible to plants [2]. The application of phosphate-based fertilizer can correct deficiency, but even when provided in a highly accessible form, as little as 10% of the phosphorus applied is recovered by plants [3], with the remainder either becoming immobilized or lost through run-off [4]. The availability of economically viable sources of phosphate fertilizer is declining [5]. There is thus some urgency associated with efforts to promote the efficiency with which crop plants are able to take up phosphate.

The concentration of inorganic phosphate (Pi) in plant tissues lies in the range 5–20 mM [6], whereas the level available in a typical soil is three orders of magnitude lower than this [7]. Thus the acquisition of Pi occurs against a steep concentration gradient [8]. Pi uptake by plants operates via both a low and a high affinity system [7,9]. The high affinity route is largely mediated by plasma membrane-localized Pi transporters belonging to the PHOSPHATE TRANSPORTER1 (Pht1) family [8,10]. Following the identification of Pi transporters in *Arabidopsis thaliana*[10], a large number of *PT* genes have been identified and characterized in a wide range of species such as *Arabidopsis thaliana*[3,11,12], rice [13-15], tomato [16], tobacco [17,18], maize [19], barley [20], *Medicago truncatula*[21], *Populus trichocarpa*[22], and soybean [23].

The chrysanthemum (*Chrysanthemum morifolium* Ramat.) is a leading ornamental species. Its productivity is usually compromised when grown in phosphate deficient soils. Here, we describe the isolation of a *Pht1* homolog present in the chrysanthemum variety ‘Nannongyinshan’, a cultivar which is relatively tolerant of phosphate deficiency [24]. The gene’s transcription profile was characterized and the beneficial effect of its constitutive expression on the plant’s ability to cope with phosphate deficiency was demonstrated.

## Methods

### Plant material and growing conditions

Cuttings of the chrysanthemum cultivar ‘Nannongyinshan’ were obtained from the Chrysanthemum Germplasm Resource Preserving Centre, Nanjing Agricultural University, China, ‘Nannongyinshan’ was a low phosphorus tolerant cultivar in our privious study, and ‘Jinba’ is a low phosphorus intolerant cultivar compared to ‘Nannongyinshan’ [24]. The cuttings were raised in a greenhouse in a 1:1 mixture of perlite and vermiculite, without any fertilizer supplementation. After two weeks, the plants were up-rooted, their roots were washed free of the rooting medium and the plants were transferred to a hydroponic solution consisting of a diluted (1:2) Hoagland’s solution [25]. The phosphate treatments were initiated one week after the transfer, by removing three plants to an aerated hydroponic solution containing either 300 (+P) or 0 (−P) μM Pi. The nutrient solution was replaced every three days. Leaf, stem and root tissue was harvested after a further 11 days, snap-frozen in liquid nitrogen and stored at −80°C.

### Isolation of *CmPT1*

The design of a pair of degenerate PCR primers (DPF/R; sequences given in Table 1) was based on a peptide alignment of the Pht1 sequences of *Arabidopsis thaliana* (GenBank accession number AED94948), *Capsicum frutescens* (ABK63958), *Lupinus albus* (AAK01938), *Hordeum vulgare* (AAN37900) and *Petunia x hybrida* (ABS12068). Total RNA was extracted from the roots of P0 treated ‘Nannongyinshan’ plants using the RNAiso reagent (TaKaRa, Japan). A full length chrysanthemum cDNA sequence (deposited in GenBank as accession KC812501) was isolated using RACE technology, following [26]. The RACE primer sequences are given in Table 1.

**Table 1:** Adaptor and primer sequences used

**Primer name**	**5′–3′ sequence**	**Usage**
Oligo d (T)_18_	TTTTTTTTTTTTTTTTTT	Reverse transcription
DPF	TGGCGGATGAAGATGCCNGARAC	Degenerated PCR
DPR	ACCGGGCGGGGAADATYTCNGC	Degenerated PCR
AAP	GGCCACGCGTCGACTAGTACGGGIIGGGIIGGGIIG	5′ -RACE
AUAP	GGCCACGCGTCGACTAGTAC	5′ -RACE
GSP5′-1	TCCGAGCAAGTGAAGG	5′ -RACE
GSP5′-2	AACCCAAATGAGTTGCTCTTGTCC	5′ -RACE
GSP5′-3	GCCATATCTTGTGCAGCTTGT	5′ -RACE
dT-B26	GACTCGAGTCAACATCGATTTTTTTTTTTTTTTTT	3′ -RACE
B26	GACTCGAGTCAACATCGA	3′ -RACE
GSP3′-1	GTGCTACCGGCGAGGTTTAT	3′ -RACE
GSP3′-2	GTCCCTGGTTACTGGTTCACTG	3′ -RACE
Full-F	ATCTCCTTTTCTTCAATCACTCTCT	ORF amplifications
Full-R	TCTAGGGTTTTATTCTTCATACCCT	ORF amplifications
*CmPT1*-EcoR-F	CCGGAATTCCGGGTTTCGAAGGAGGAGTGTATCCATG	Functional complementation
*CmPT1*-Not-R	ATTTGCGGCCGCAACAGTATTCAGTCAATCAAGCCGG	Functional complementation
qGSP-F	CTCGCTCGTTGTTCTTGGTATC	qRT-PCR
qGSP-R	AACAGTATTCAGTCAATCAAGC	qRT-PCR
EF1A-F	TTTTGGTATCTGGTCCTGGAG	qRT-PCR
EF1A-R	CCATTCAAGCGACAGACTCA	qRT-PCR
35S-F	AGATACAGTCTCAGAAGACCAAAGG	Transgenic detection
35S-R	TTGATATTCTTGGAGTAGACGAGAG	Transgenic detection

### Sequence analysis

The open reading frame (ORF) of the full length cDNA isolated from ‘Nannongyinshan’ was identified using the ORF finder program (DNASTAR. Lasergene. v7.1). The location of hydrophobic and putative transmembrane domains was enabled through the software package mounted at http://expasy.org/tools/protscale.html. Multiple peptide alignments were carried out using DNAman software (v5.2.2.0; Lynnon Biosoft, St Louis, QC, Canada), and phylogenetic analyses using Clustal X and MEGA v4.0 software.

### *CmPT1* transcription profiling

Total RNA was extracted from root, stem and leaf tissue of plants grown both in the + P and -P treatments using the RNAiso reagent, and was then used as the template for real-time quantitative PCR (qRT-PCR) assays, based on the SYBR Green master mix (SYBR *Premix Ex Taq*™ II, TaKaRa Bio) and the gene-specific primer pair qGSP-F/-R (sequences given in Table 1), which generated a 163 bp fragment. The reference sequence was a 151 bp fragment of *EF-1a*, amplified with the primers EF1a -F/-R (sequences given in Table 1). Each 20 μl RT-qPCR contained 10 μl SYBR Green master mix, 10 ng cDNA template and 0.2 μM of each primer. The PCR program comprised an initial denaturation step (95°C/60 s), followed by 40 cycles of 95°C/15 s, 60°C/15 s, 72°C/45 s. Relative transcription levels were estimated using the 2^-ΔΔ*Ct*^ method [27].

### Complementation of a yeast mutant strain defective for Pi uptake

The function of the chrysamthemum gene (denoted *CmPT1*) was studied by its heterologous expression in yeast strain MB192 (*MAT*a *pho3-1 Dpho84::HIS3 ade2 leu2-3.112 his3-532 trp1-289 ura3-1.2 can1*) which lacks phosphate transporter activity. First, the *CmPT1* ORF was amplified using a Phusion High-Fidelity PCR kit (New England Biolabs, lpswich, MA, USA) based on the primer pair *CmPT1*-EcoR-F/Not-R (sequences given in Table 1), and the resulting amplicon was digested with *Eco*RI and *Not*I and then introduced into the yeast expression vector p112A1NE. The structure of the resulting recombinant plasmid (p112A1NE-*CmPT1*) was confirmed by restriction enzyme digestion and DNA sequencing. MB192 cells were transformed with either p112A1NE-*CmPT1* or with the empty p112A1NE vector. Transgenic cells were grown in a yeast nitrogen base (YNB) medium until up to the logarithmic phase, and the medium was then replaced with a range of Pi concentrations (20, 60 and 100 μM) and the cells left to grow for a further 20 h. Bromocresol Purple was used to indicate the pH of the medium, giving a color shift, from yellow to purple, during the acidification of the liquid medium: this change correlated well with the growth of the yeast cells [14]. Thereafter, the cells were transferred into YNB medium containing 60 μM Pi for a further 40 h. The optical density of the yeast cultures was measured every 8 h. The pH dependence of Pi uptake was studied by growing the cells in a series of MES-based YNB buffers containing 60 μm Pi at a pH of between 4 and 8. The kinetic properties of CmPT1 were analysed by feeding the transformed yeast cells with ^32^P labelled Pi, following the methods described by [13]. The *K*_*m*_ value was calculated using GraphPad v4.0 software.

### Construction of an constitutive expression vector and the development of transgenic plants

*CmPT1* was inserted into the pBIG vector (offered by Professor Junping Gao, Department of Ornamental Horticulture, China Agricultural University) under the control of the CaMV 35S promoter. The structure of the resulting pBIG-*CmPT1* recombinant plasmid was confirmed by restriction enzyme digestion and DNA sequencing, and was then transformed into *Agrobacterium tumefaciens* strain EHA105 via the heat shock method [28]. The recipient of the transgene was the chrysanthemum cultivar ‘Jinba’, which is intolerant of phosphate deficiency [24]. Transformation was effected using a slightly modified form of the procedure described by [29]. Explants developing multiple shoots were transferred to a selective regeneration medium, with the addition of 1.0 mg · L^-1^ 6-BA, 0.5 mg · L^-1^ NAA, 10 mg · L^-1^ kanamycin and 300 mg · L^-1^ carbenicillin. Regenerating shoots were then rooted by transferring the material to a half-strength Murashige and Skoog medium containing 7.5 mg · L^-1^ kanamycin and 200 mg · L^-1^ carbenicillin. After acclimation, kanamycin-resistant plants were transplanted to soil and grown on in a greenhouse.

### The phenotype of transgenic lines constitutively expressing *CmPT1*

Verification of transformation was obtained by a PCR assay of genomic DNA isolated from both presumptively transformed rooted plants, primed with the oligonucleotide pair 35 s-F/-R (sequences given in Table 1) which targets the CaMV 35S promoter sequence. The PCR was initiated by a denaturation step (94°C/5 min, which was followed by 30 cycles of 94°C/45 s, 55°C/45 s, 72°C/30 s, and completed with a final elongation step of 72°C/10 min. Transcript abundance of *CmPT1* in the confirmed primary transformants was obtained by qRT-PCR, as described above. Two independent transgenic plants (CmPT1-Oe1 and CmPT1-Oe2), along with non-transformed ‘Jinba’ plants were exposed to either Pi-sufficient (300 μM Pi; +P) and P-deficiency (15 μM Pi; -P) conditions for 20 days. Each assay involved three replicates of a set of ten plants per genotype. Plant height, root volume, total dry biomass and the phosphate concentration in the whole plant were measured. The root volume was obtained by displacement [30]. Dry weight was obtained by weighing plants held at 80°C for three days. The whole plant phosphate concentration was estimated from a ~0.1 g sample of dry matter, following [31].

## Results

### *CmPT1* encodes a Pht1 Pi transporter

**Figure 1:**
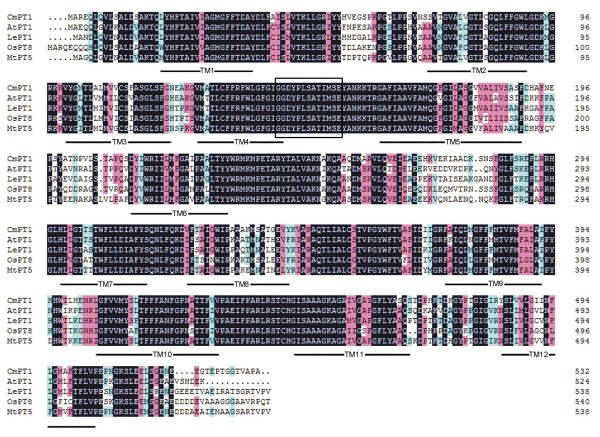
**Alignment of the peptide sequences of known high affinity Pi transporters present in *A. thaliana *(AtPT1, AED94948), tomato (LePT1, AAB82146), rice (OsPT8, Q8H6G8) and *M. truncatula *(MtPT5, ABM69111).** Identical peptides are highlighted in black, and conservative substitutions in pink. Putative CmPT1 transmembrane domains are shown underlined. The Pht1 signature sequence is shown boxed.

**Figure 2:**
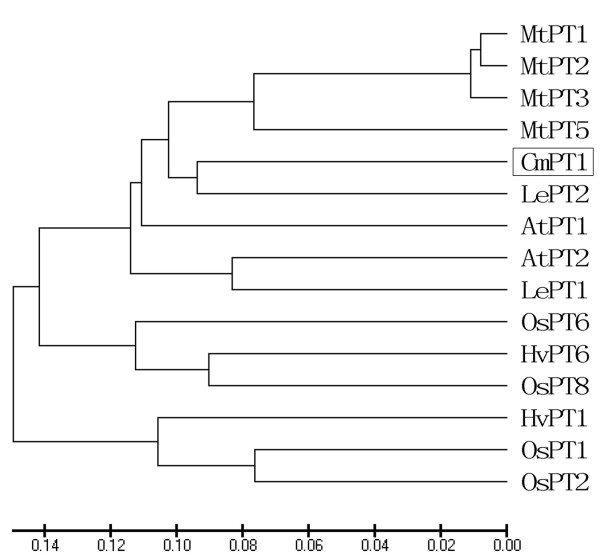
**Phylogenetic relationships between CmPT1 and other plant Pi transporters.** AtPT1 (AED94948) and AtPT2 (AAC79607) from *A. thaliana*; HvPT1 (AAO32938) and HvPT6 (AAN37901) from barley, MtPT1 (AAB81346), MtPT2 (AAB81347), MtPT3(ABM69110) and MtPT5 (ABM69111) from *M. truncatula*; LePT1 (AAB82146), LePT2 (AAB82147) from tomato, OsPT1 (Q8H6H4), OsPT2 (Q8GSD9), OsPT6 (Q8H6H0) and OsPT8 (Q8H6G8) from rice.

The full-length *CmPT1* cDNA was a 1,875 bp sequence, consisting of a 1,599 bp ORF, an 87 bp 5′-UTR and a 189 bp 3′-UTR (Figure 1). Its predicted translation product was a 532 residue polypeptide with a calculated molecular mass of 57.97 kDa and a pI of 8.60. The protein contains 12 transmembrane domains with a large hydrophilic loop between TM6 and TM7 (Figure 1). The peptide sequence shares a high level of homology with known high affinity plant Pht1 proteins: 77.1% with AtPT1 (GenBank accession number AED94948), 78.5% with LePT1 (AAB82146), 71.8% with OsPT8 (Q8H6G8) and 78.9% with MtPT5 (ABM69111) (Figure 1). A phylogenetic analysis showed that the chrysanthemum sequence is closely related with other plant Pi transporters, and particularly with its tomato homolog LePT2 (AAB82147) (Figure 2). The Pht1 signature sequence GGDYPLSATIxSE [32] is present in CmPT1.

### Transcription of *CmPT1*

**Figure 3:**
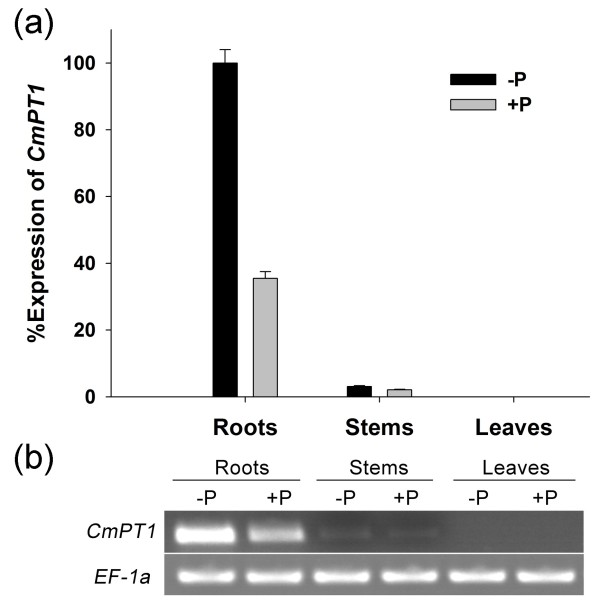
**Transcription of *CmPT1 *in response to Pi availability. (a)** Relative quantification and **(b)** semi-quantitative measurements of transcript abundance in the root, stem and leaf of plants grown at + P (300 μM Pi) and -P (0 μM Pi)*.*

*CmPT1* transcription was strong in the root, weak in the stem, and below the level of detection in the leaf of plants exposed to either under both Pi-sufficient (300 μM Pi; +P) and Pi-deficient (0 μM Pi; -P) conditions (Figure 3). The relative transcript abundance in the root of + P and -P plants was, respectively, 35.5 and 100.0, and in the stem, respectively 2.1 and 3.1.

### Complementation test in yeast

**Figure 4:**
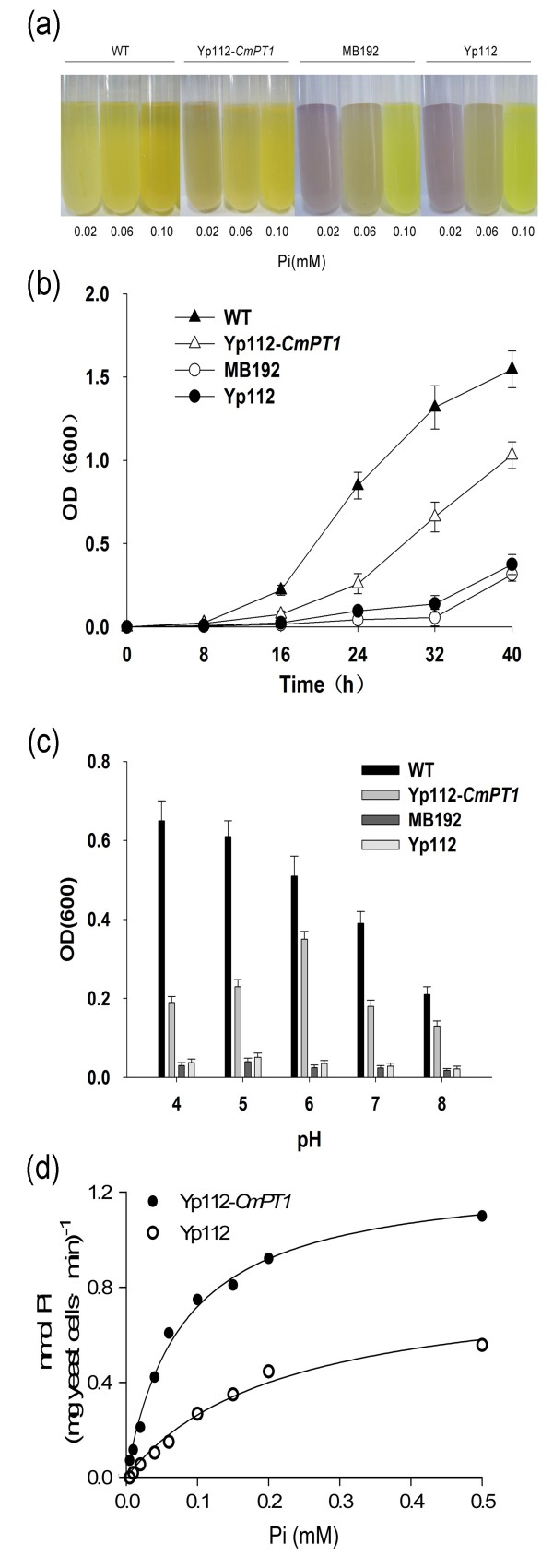
**Complementation assay of *CmPT1 *in yeast strain MB192. (a)** Test for acid phosphatase activity. Yp112-*CmPT1* carries the *CmPT1* transgene, Yp112 is an empty vector, WT is wild type MB192. **(b)** Growth of MB192, Yp112-*CmPT1*, Yp112, and WT cells after 40 h growth in the presence of 60 mM Pi. **(c)** The effect of varying the pH of the medium on the growth of MB192, Yp112-*CmPT1*, Yp112 and WT. **(d)**^32^P transport in Yp112-*CmPT1* as a function of Pi concentration. A non-linear regression of Pi uptake by Yp112-*CmPT1* at pH6 was used to estimate the *K*_*m*_ of Pi uptake. OD(600): optical density measured at 600 nm.

Yeast strain MB192 cells heterologously expressing *CmPT1* (Yp112-*CmPT1*) were able to survive at 0.02 mM Pi, and grew well at 0.06 mM Pi; neither wild type (WT) cells, nor MB192 cells containing only Yp112 could do so. At 0.1 mM Pi, all cell types grew equally well (Figure 4a, b). Both WT and Yp112-*CmPT1* cells grew faster at pH 4–6 than at 7–8 (Figure 4c). The pH optimum for Yp112-*CmPT1* was around 6, whereas for the WT it was 4–5. The results are consistent with the notion that CmPT1 operates as an H^+^/H_2_PO_4_ symporter. To exclude interference from the activity of other PTs (particularly the low affinity ones), the dynamics of Pi transport of cells transformed with either p112A1NE-*CmPT1* or the empty vector was monitored at 0.5 mM Pi. The estimated mean *K*_*m*_ of CmPT1 was 35.2 μM Pi, as determined from three independent experiments (Figure 4d). Thus, CmPT1 is likely a high Pi affinity transporter, mediating Pi uptake in the micromolar range.

### *CmPT1* constitutive expression in chrysanthemum enhances tolerance to Pi deficiency

**Figure 5:**
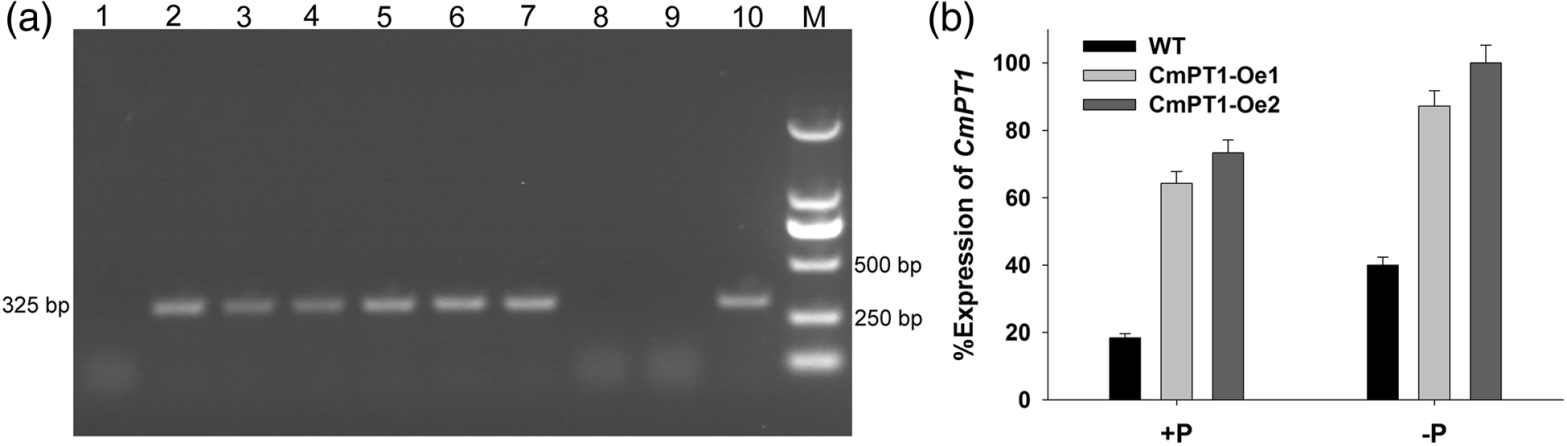
**Validation of transgenic plants. (a)** PCR analysis of genomic DNA extracted from kanamycin resistant regenerants. **(b)** Relative *CmPT1* transcript abundance in wild type and transgenic plants.

**Figure 6:**
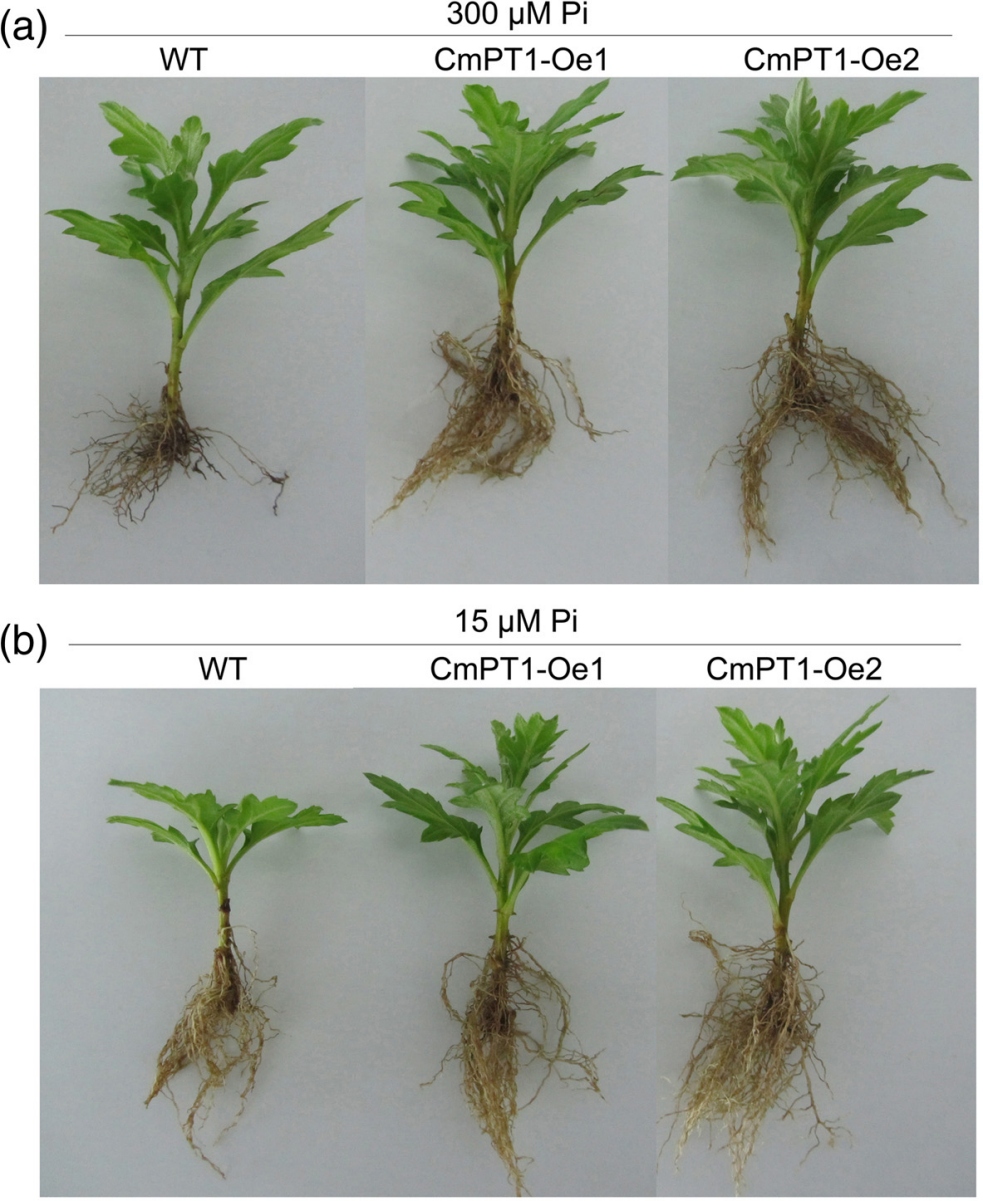
**The effect on growth of constitutively expressing *CmPT1.*** Wild type (‘Jinba’) and *CmPT1* constitutive expression transgenics grown in the presence of **(a)** sufficient Pi (“+P”) and **(b)** deficient Pi (“-P”) conditions.

**Figure 7:**
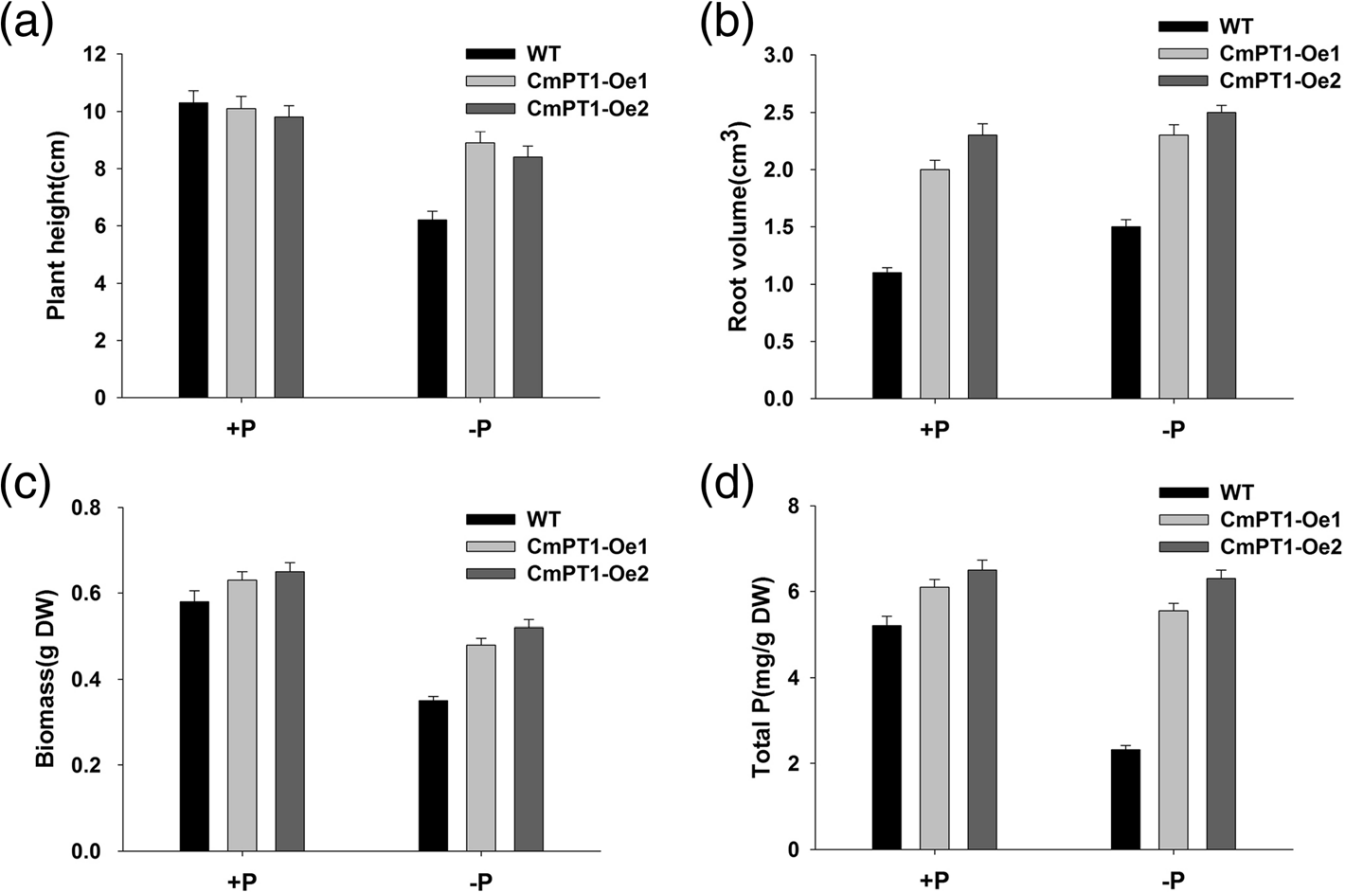
**Phenotypic differences of the wild-type and *CmPT1*-overexpression lines.** The effect of *CmPT1* constitutive expression on **(a)** plant height, **(b)** root volume, **(c)** biomass accumulation and **(d)** phosphate accumulation. The data are expressed as means ± standard error (*n* = 5). Columns bearing the same letter did not differ significantly from one another at P < 0.01.

Six independent rooted presumptive *CmPT1* transformants were selected by the genomic PCR assay (Figure 5a). When subjected to qRT-PCR, it was clear that the level of *CmPT1* transcription in CmPT1-Oe1 and CmPT1-Oe2 was significantly higher than in the wild type plant both at Pi-sufficient (300 μM Pi; +P) and P-deficiency (15 μM Pi; -P) conditions (Figure 5b). The effect *CmPT1* constitutive expression was to promote root growth and increase the accumulation of phosphate at + P (Figures 6 and 7), while at -P, the plant height of the two transgenic plants was increased by respectively 38.7% and 35.5% over that of the wild type (Figure 7a); similarly, root volume in both transgenics was enhanced by, respectively, 53.3% and 66.7% (Figure 7b), dry weight by 37.1% and 48.6% and phosphate concentration by 54.4% and 68.7% (Figure 7c, d). Thus the constitutive expression of *CmPT1* helped chrysanthemum plants to cope with phosphate deficiency.

## Discussion

The uptake of Pi from a heterogeneous, constantly fluctuating environment is a major challenge faced by all plants. The process is largely governed by Pht1 transporters [2]. The predicted product of the chrysanthemum *Pht1* homologue extracted from the cultivar ‘Nannongyinshan’ forms 12 transmembrane domains, separated into two groups of six by a charged hydrophilic loop (Figure 1). This structural arrangement is typical for Pht1 transporters [6]. The CmPT1 sequence shares a high level of sequence identity to other known high affinity Pi transporters and includes the signature sequence GGDYPLSATIxSE. A number of members of the Pht1 family are strongly expressed in the root, and are induced by phosphate starvation [10,13,14,16,33], just as was the case for *CmPT1* (Figure 3a, b). Nevertheless its pattern of transcription is not completely the same as that of certain high affinity Pi transporters, in particular those of *LePT1*, which is transcribed in both the root and the leaf of tomato [16], and *OsPT8* which is also transcribed in the rice grain [14]. *CmPT1* transcript abundance was below the level of detection in the leaf, even in plants growing under phosphate deficient growing conditions, while its level in the stem was low but still discernible. The evidence as a whole strongly suggests that CmPT1 acts as a Pi transporter in chrysanthemum.

Several approaches have been used to investigate the Pi transport properties of the various PHT1 family members. In our study, CmPT1 was able to complement the loss of high affinity Pi transporter activity of the yeast mutant MB192 in the high affinity concentration range (Figure 4). Its estimated *Km* of 35.2 μM (Figure 4d) lies within the *K*_*m*_ range of other plant high affinity phosphate transporters, measured by a similar method – the range being from 13 μM to 97 μM [13,14,21]. Heterologous expression in yeast provides an informative means of characterizing plant proteins, but the system does have some limitations with respect to assessing the kinetics of plant phosphate transporters, the yeast complementation only partially reflects the behavior of PHT1 proteins in plants [2,20]. Estimates of *K*_*m*_ obtained by transcription in suspension cultures are substantially lower: 3.1 μM for *A. thaliana* Pht1 when transcribed in tobacco cells [34]; 9.1 μM for the barley protein HORvu;Pht1;1 when expressed in rice cells [20]. It may be possible to obtain a more accurate estimate of *Km* to CmPT1 by using a homologous system in the future.

Transcription of the gene encoding the primary transporter is often considered to be the control point in pathways of nutrient uptake [35]. Many efforts have been made to research the function of the *PT* genes and the possibility of using the *PT* genes to improve the adaptability to Pi-deficient stress of crops. Overexpression of an *Arabidopsis thaliana* high-affinity phosphate transporter gene in tobacco cultured cells enhances cell growth under phosphate-limited conditions [34]. Suspension cells of transgenic rice that over-expressed a high-affinity phosphate transporter *HvPT1* were able to take up phosphate at a much higher rate than control cells [20], but over-expression of the same gene in transgenic barley plants does not enhance phosphate uptake rates at the whole plant level [36]. Park transferred a high-affinity phosphate transporter gene from tobacco (*NtPT1*) to rice enhanced phosphate uptake and accumulation under low-P condition [37]. Over-expressing low affinity phosphate transporter genes *OsPT1* and *OsPT2* in rice cannot bring significant differences to the transgenic plants under Pi-deficient supply [15,38]. Here, a Pht1 transporter gene which cloned from a low P tolerant cultivar of cut chrysanthemum ‘Nannongyinshan’ transferred to ‘Jinba’, a low P sensitive cultivar, the growth of transgenic plants was significantly improved over that of the non-transformed ones under Pi deficient conditions (Figures 6 and 7). Considering the two cultivars sharing the same sequence of *CmPT1* and the transgenic and non-transgenic plants having same genetic background, physiological and biochemical changes are direct results of the overexpression of *CmPT1* gene. Two possibilities can be envisaged to explain mechanisms of these changes. More copies of the transporter protein are inserted into the plasma membrane in the overexpression transgenic lines. Evidence for the transcriptional control of PT activity has been shown by parallel increases in PT transcript and protein levels detected by high-affinity PT antibody in tomato [39]. The *CmPT1* gene may participate in the Pi-starvation signal transduction pathways, Overexpression of the *CmPT1* gene directly or indirectly caused Pi-dependent root architecture alteration with enhanced root elongation and proliferated lateral root growth (Figures 6 and 7b), these responses enable the optimization of soil exploration and of the absorptive surface area of the root [40].

## Conclusion

In conclusion, *CmPT1* encodes a typical, root-expressed, high affinity phosphate transporter in chrysanthemum. Taking the flexible expression pattern into account, CmPT1 might function in a wide range of Pi environments and play a significant role in phosphate acquisition under natural conditions.

## Competing interests

The authors declare that they have no competing interests.

## Authors’ contributions

PL and AS performed the experiments; PL wrote the manuscript; SC, SZ, ZG and JJ edited the manuscript; WF, SC, YL and JJ revised the manuscript; JJ and FC designed the experiments. All authors read and approved the final manuscript.
